# HIV-1/HAART-Related Lipodystrophy Syndrome (HALS) Is Associated with Decreased Circulating sTWEAK Levels

**DOI:** 10.1371/journal.pone.0144789

**Published:** 2015-12-14

**Authors:** Miguel López-Dupla, Elsa Maymó-Masip, Esteban Martínez, Pere Domingo, Manuel Leal, Joaquim Peraire, Consuelo Viladés, Sergi Veloso, Mireia Arnedo, Sara Ferrando-Martínez, Raúl Beltrán-Debón, Verónica Alba, Josep Mª Gatell, Joan Vendrell, Francesc Vidal, Matilde R. Chacón

**Affiliations:** 1 Hospital Universitari Joan XXIII. IISPV, Universitat Rovira i Virgili, Tarragona, Spain; 2 Centro de Investigación Biomédica en Red de Diabetes y Enfermedades Metabólicas (CIBERDEM), Tarragona, Spain; 3 Hospital Clinic, Universitat de Barcelona, Barcelona, Spain; 4 Hospital de la Santa Creu i Sant Pau, Universitat Autònoma de Barcelona, Barcelona, Spain; 5 Hospital Universitario Virgen del Rocio, IBIS, Universidad de Sevilla, Sevilla, Spain; University of Malaya, MALAYSIA

## Abstract

**Background and Objectives:**

Obesity and HIV-1/HAART–associated lipodystrophy syndrome (HALS) share clinical, pathological and mechanistic features. Tumor necrosis factor-like weak inducer of apoptosis (TWEAK) is a multifunctional cytokine that plays an important role in obesity and related diseases. We sought to explore the relationship between HALS and circulating levels of soluble (s) TWEAK and its scavenger receptor sCD163.

**Methods:**

This was a cross-sectional multicenter study of 120 HIV-1-infected patients treated with a stable HAART regimen; 56 with overt HALS and 64 without HALS. Epidemiological and clinical variables were determined. Serum levels of sTWEAK and sCD163 levels were measured by ELISA. Results were analyzed with Student’s *t*-test, Mann-Whitney U and χ^2^ test. Pearson and Spearman correlation were used to estimate the strength of association between variables.

**Results:**

Circulating sTWEAK was significantly decreased in HALS patients compared with non-HALS patients (2.81±0.2 vs. 2.94±0.28 pg/mL, p = 0.018). No changes were observed in sCD163 levels in the studied cohorts. On multivariate analysis, a lower log sTWEAK concentration was independently associated with the presence of HALS (OR 0.027, 95% CI 0.001–0.521, p = 0.027).

**Conclusions:**

HALS is associated with decreased sTWEAK levels.

## Introduction

Several morphological and mechanistic events observed in HIV-1/Highly Active Antiretroviral Therapy (HAART)–associated lipodystrophy syndrome (HALS), an acquired form of lipodystrophy, are reminiscent of those seen in obesity[1]. This notion has been consistently demonstrated with respect to the disequilibrium of several adipokines/cytokines, such as leptin, adiponectin, resistin, RBP4, vaspin, visfatin and FABP4, among others[2–4].

Tumor necrosis factor-like weak inducer of apoptosis (TWEAK) is a multifunctional cytokine of the TNF superfamily that exists in two forms, a membrane anchored form (mTWEAK) and a cleaved soluble form (sTWEAK)[5]. TWEAK is expressed by natural killer cells, macrophages, and dendritic cells[6,7], and regulates a diverse range of cellular processes including proliferation, differentiation, migration, cell survival, and apoptosis. TWEAK has also been shown to act as a proangiogenic and proinflammatory factor[7]. Despite the inflammatory capacity described for TWEAK, a competitive interfering activity with TNFα signaling has been defined in several settings[8–11], resulting in a modulatory effect over the adverse inflammatory and metabolic effects of TNFα.

Circulating sTWEAK concentrations have been proposed as an independent biomarker of cardiovascular disease[12–16]. Reduced sTWEAK levels have been detected in obesity and associated metabolic diseases. sTWEAK can be modulated, in part, by the scavenger receptor CD163 (cluster of differentiation 163). CD163 is exclusively expressed in monocytes and macrophages [17], but is also released in the circulation as a soluble form (termed sCD163) following pro-inflammatory stimulation by LPS [18][19], and is a specific plasma/serum marker for macrophage/monocyte activity. CD163 can bind to hemoglobin-haptoglobin (Hb-Hp) complexes, triggering an anti-inflammatory response such as the release of IL-10 [20]; however, the recognition of bacteria by CD163 can result in amplification of pro-inflammatory cytokine production, including TNFα, IL-1β and IL-6 by mononuclear phagocytes, a response similar to that seen during TLR activation[21]. Although the function of sCD163 is not clear, a role in the elimination of *Staphylococcus aureus* has recently been described [22] in addition to the findings of anti-inflammatory effects through inhibition on T lymphocyte activation and proliferation [23].

It has been postulated that macrophages can recognize and internalize sTWEAK, thereby decreasing its plasma/serum concentration[24,25]. High circulating levels of sCD163 have been linked with a more pro-inflammatory profile in obesity[26], and have been associated with atherosclerosis in HIV-infected patients[27,28]. In the HIV setting, a small cohort study of HIV-infected patients reported reduced sTWEAK and increased sCD163 circulating levels, but no changes in sTWEAK levels after 48 weeks on HAART[29]. A particularly interesting subset of patients with HIV are those with an associated lipodystrophy syndrome, termed HALS. These patients have an increased risk of cardiovascular disease due to increased inflammation and persistent immune activation. Identification of biomarkers in these patients may improve cardiovascular risk predictions by traditional stratification scales. To gain insight into the role of sTWEAK and sCD163 in patients with HALS and its associated metabolic derangements, we investigated sTWEAK serum concentrations in a well characterized cohort of Caucasian Spanish treated HIV-infected patients with and without HALS, in relation to immunovirological, inflammatory and metabolic parameters.

## Patients and Methods

### Design, setting and participants

This was a multi-center, cross-sectional, case-control study comprising 120 adult treated HIV-1-infected patients, 64 without HALS and 56 with overt HALS. The patients were from a large cohort comprising 558 adult treated HIV-1-infected patients (318 without HALS and 240 with overt HALS) that has participated in our genetic and molecular studies of HALS pathogenesis[2]. In the present analysis, we used the 120 patients for whom stored serum and plasma samples, drawn at enrollment, were available. No serum or plasma samples were available for the remaining 438 patients (they have been exhausted through studies). Patients were consecutively recruited between 2004 and 2006 at the HIV outpatient clinic of the participating hospitals: Hospital de la Santa Creu i Sant Pau, Barcelona; Hospital Clinic, Barcelona; Hospital Virgen del Rocio, Sevilla; and Hospital Joan XXIII, Tarragona, which included 32, 28, 20 and 40 patients, respectively. All are tertiary university-affiliated hospitals located in Spain. Patients were selected from among those who were receiving HAART, defined as the combination of two nucleoside reverse transcriptase inhibitors (NRTI) plus either a non-nucleoside reverse transcriptase inhibitor (NNRTI) or a protease inhibitor (PI). All the selected patients fulfilled the following inclusion criteria: age >18 years, presence of HIV-1 infection, stable HAART regimen for at least 1 year and presence or absence of HALS according to standardised criteria that we have previously reported [4,30,31]. Exclusion criteria were the presence of active opportunistic infections, current inflammatory diseases or conditions, consumption of drugs with known metabolic effects, type 2 diabetes mellitus, acute or chronic renal failure, pregnancy, history of vaccination during the last year and plasma C-reactive protein >1mg/dL.

All participants provided their written informed consent to participate in this study. The ethics committees from each participating center approved this consent procedure. The study was reviewed and approved by the ethics committee from Hospital Universitary de Tarragona Joan XXIII, Hospital Clinic de Barcelona, Hospital de Santa Creu i Sant Pau de Barcelona and Hospital Universitario Virgen del Rocio, de Sevilla before the study began.

### Assessment of HALS

All patients were given a full physical examination to assess the type (lipoatrophy, lipohypertrophy or mixed) and degree (slight, moderate or severe) of lipodystrophy. Criteria for lipoatrophy were one or more of the following: loss of fat from the face, arms and legs, prominent veins in the arms and legs and thin buttocks. Lipohypertrophy was defined by the presence of one or more of the following criteria: increase in abdominal perimeter, breast and/or neck fat deposition. We defined mixed lipodystrophy as present when at least one characteristic of lipoatrophy and one of lipohypertrophy were concomitantly present in a given patient. Lipodystrophy was categorized in accordance with a previously validated scale [32]: nil {0}, slight {1}, moderate {2} and severe {3}. Doubtful cases were excluded. This categorization was evaluated in the face, arms, legs, buttocks, abdomen, neck and breasts. The sum of the values corresponding to each body area indicated the degree of lipodystrophy: nil {0}, slight {1–6}, moderate {7–12} and severe {13–18} [2–4,31–32]. In the present study, we included only extreme lipodystrophy phenotypes (nil vs. severe cases) in order to avoid superposition between groups. To objectively assess the distribution of visceral adipose tissue (VAT) and subcutaneous adipose tissue (SAT), a single-slice CT scan was performed at the level of L4 in the 558 patients included in this study. The surface of adipose tissue was measured in cm^2^.

### Laboratory methods

#### Collection of blood samples

Blood was drawn from a peripheral vein after overnight fast. Whole blood was used to determine CD4^+^ T-cell count and to isolate DNA. Serum was obtained by centrifugation and stored at -80°C until used.

#### HIV-1 infection-related parameters

HIV-1 infection was diagnosed by a positive EIA (CHIV Advia Centaur, Siemens® HealthCare Diagnostics, TarryTown, NY, USA) and confirmed by western blotting (INNO-LIA HIV I/II SCORE, Fujorebio Europe ®, Gent, Belgium). Plasma HIV-1 viral load was determined with the COBAS® Ampliprep / COBAS® TaqMan v.2.0 Roche® Diagnostics GmbH, Mannheim Germany). CD4^+^ T-cell count was analyzed in a FAC Scan flow cytometer (Becton Dickinson, San Jose, CA, USA).

#### Blood chemistry

Concentrations of glucose, insulin, total cholesterol, high-density lipoprotein cholesterol (HDL-cholesterol), low-density lipoprotein cholesterol (LDL-cholesterol) and triglycerides were measured using standard enzymatic methods. The homeostatic model assessment insulin resistance index (HOMA-IR) was calculated with the following formula: (insulin (μIU/mL) x glucose (mmol/L)/22.5).

#### sTWEAK, sCD163, omentin and sCD14 circulating levels

Serum concentrations of sTWEAK and sCD163 were determined by ELISA using the commercially available human TWEAK/TNFSF12 Kit DY1090, and human CD163 Kit DY1607 (R&D Systems Europe, Abingdon, Oxon, United Kingdom), respectively. The intra- and inter-assay coefficients of variation were 2.5 and 7.0% for sTWEAK and 2.4 and 6.4% for sCD163, respectively. Plasma omentin concentration was determined using a commercial omentin-1 human ELISA (BioVendor GmbH, Heidelberg, Germany). Plasma sCD14 was measured with a commercially available ELISA kit on sera diluted 0.01% (R&D Systems, Abingdon, UK). All ELISA assays were performed in duplicate.

### Statistical analysis

All variables were examined for their distribution characteristics. Normally distributed data was expressed as mean ± standard deviation (SD), whereas variables with a skew distribution were represented as the median (25^th^percentile–75^th^percentile) or transformed using the logarithm function (sTWEAK and sCD163, among others). Categorical variables were expressed as number (percentage). Qualitative variables were analyzed by the χ^2^ test or Fisher’s exact test, where necessary. Student’s *t*-test was used to compare continuous variables between 2 groups. We used the Mann-Whitney U test to compare variables that did not fit a Gaussian distribution. Associations between quantitative variables were evaluated by Pearson correlation analysis or Spearman correlation for non-normally distributed variables. The independence of the observed associations with sTWEAK and sCD163 serum levels was evaluated by linear regression analysis. Logistic regression test was performed to analyze the predictors of HALS. Differences with a p value <0.05 were considered significant for all statistical tests. The statistical studies were performed using the SPSS statistics for Windows (Version 17.0; Chicago: SPSS Inc. USA).

## Results

### Characteristics of the studied Cohort

Demographic and clinical characteristics of the patients studied categorized according to the presence or absence of HALS are presented in Table 1. Note that the gender distribution with a marked male predominance reflects the epidemiologic distribution of HIV infection in Spain. All patients from the HALS group had an extreme lipodystrophy phenotype that included marked lipoatrophy of enough severity to be treated with facial implants. HALS patients were slightly older than non-HALS patients, but this and the gender distribution was not significantly different between groups (mean age 52.16±8.04 and 49.44±8.86, respectively). The prevalence of hepatitis C virus (HCV) infection was equal between groups. Intravenous drug users were more frequent in the HALS group. The duration of HIV infection was significantly longer in the HALS group (p = 0.008). CD4^+^ T-cell counts were non-significantly different between groups. Concerning HIV-1 viral load, non-HALS patients had greater plasma viral load than HALS patients (2.46±1.16 vs 1.86±0.53, p = 0.001). Concerning antiretroviral drug use, data of the type of drugs that patients had received and time of exposure are presented in detail in Table 1. The only difference between groups was a significant increase use of d4T (stavudine) by HALS patients (p = 0.02).

**Table 1:** Demographic and clinical characteristics of the patients studied, categorized according to the presence or absence of HALS.

		Non-HALS N = 64	HALS N = 56	*p* value
**Age (years)**		49.44±8.86	52.16±8.04	0.08
**Sex male/ n (%)**		50 (78.1)	51 (91.1)	0.08
**HCV infection, n (%)**		14 (21.9)	17 (30.4)	0.29
**HIV risk group, n (%)**	**Homosexual**	42 (65.6)	29 (51.8)	0.14
	**Heterosexual**	15 (23.4)	11 (19.6)	0.66
	**Injection drug user**	7 (10.9)	16 (28.6)	0.02
	**Other/Unknown**	0	0	
**Duration of HIV infection (years)**		15.46±4.4	17.85±5.03	0.008
**CD4 cell count (cells/ml)**		717.08±217.92	643.27±301	0.14
**Log plasma HIV load (copies/ml)**		2.46±1.16	1.86±0.53	0.001
**Duration of HAART (months)**		143.63±48.38	149.36±58.96	0.56
**Exposure to NRTI before HAART, yes, n (%)**		64 (100)	56 (100)	1
**NRTI, n (%)**		64 (100)	55 (98.2)	0.47
**Cumulative time on NRTI (months)**		156 (120–192)	180 (132–192)	0.39
**NNRTI, n (%)**		49 (76.6)	42 (75)	1
**Cumulative time on NNRTI (months)**		36 (24–93)	60 (33–96)	0.22
**PI, n (%)**		54 (84.4)	51 (91.1)	0.41
**Cumulative time on PI (months)**		84 (45–132)	96 (72–132)	0.2
**AZT, n (%)**		36 (56.3)	36 (64.3)	0.46
**Cumulative time on AZT (months)**		24 (12–60)	42 (24–60)	0.3
**d4T, n (%)**		27 (42.2)	36 (64.3)	0.02
**Cumulative time on d4T (months)**		42 (24–72)	36 (24–60)	0.73

Data are expressed as mean ± standard deviation or as median and IQ 25–75%. Qualitative variables are expressed as percentages. BMI: Body mass index. HALS: HIV/antiretroviral treatment lipodystrophy syndrome. HCV: hepatitis C virus. HAART: Highly active antiretroviral treatment. NRTI: Nucleoside reverse transcriptase inhibitor. NNRTI: Non-nucleoside reverse transcriptase inhibitor. PI: Protease inhibitor. AZT: zidovudine. d4T: stavudine

### sTWEAK and sCD163 serum levels in HALS. Relationship with metabolic parameters, immunovirologic status and pro- and anti-inflammatory biomarkers

**Fig 1:**
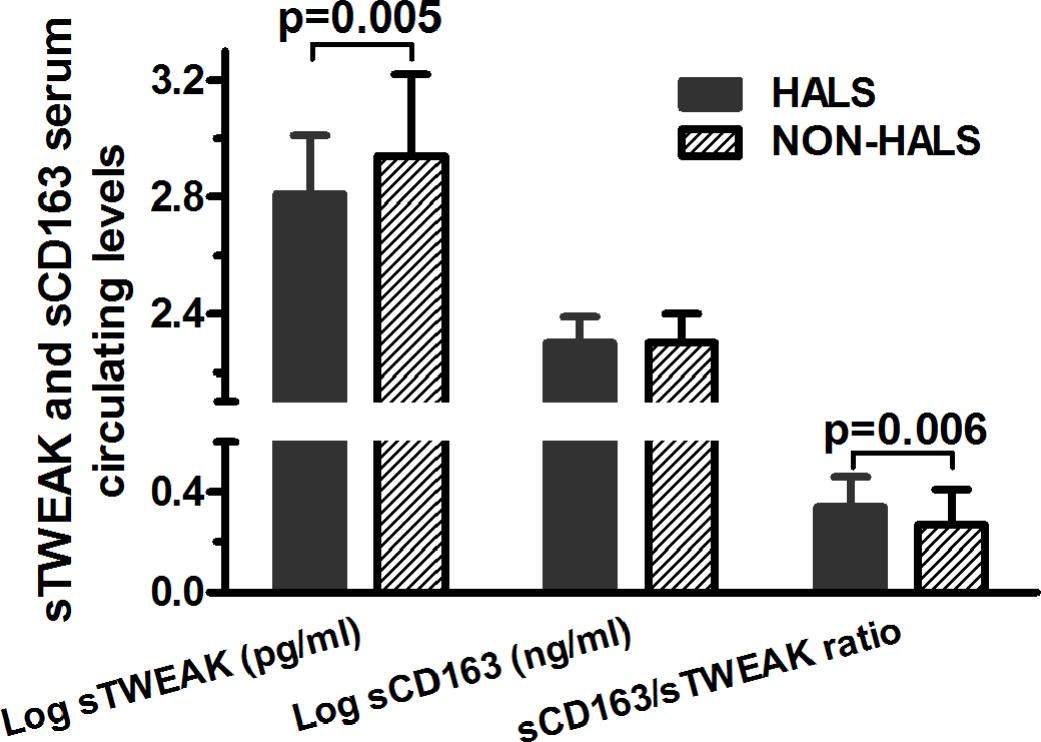
Serum sTWEAK, sCD163 levels, and sCD163/sTWEAK ratio in patients with and without HALS. Serum concentration of sTWEAK and sCD163 and sCD163/sTWEAK ratio are log transformed and are expressed as mean ± standard deviation.

Log sTWEAK circulating levels were significantly reduced in HALS patients as compared with non-HALS patients (Fig 1, Table 2), however no differences were found with respect to log sCD163 levels between groups. The sCD163/ sTWEAK ratio was significantly increased in HALS patients (Fig 1, Table 2). Concerning metabolic parameters, plasma triglycerides, total cholesterol, LDL-cholesterol, HDL-cholesterol, insulin and HOMA-IR were similar between groups (Table 2). Concerning pro- and anti-inflammatory biomarkers, circulating sCD14 and omentin levels were significantly lower in HALS patients (p<0.001 for both) (Table 2).

**Table 2:** Relationship between metabolic parameters and other cytokines and the presence or absence of HALS.

	Non-HALS N = 64	HALS N = 56	*p* value
**Total cholesterol (mmol/L)**	5.08±1.15	5.33±1.24	0.26
**LDL cholesterol (mmol/L)**	3.21±0.89	3.39±1	0.32
**HDL cholesterol (mmol/L)**	1.08±0.25	1.1±0.29	0.67
**Triglycerides (mmol/L)**	2.31±1.73	2.64±2.26	0.37
**Glucose (mmol/L)**	5.89±2.05	5.79±2.05	0.79
**HOMA-IR**	7.2±4.3	7.94±9.98	0.61
**Insulin (μU/mL)**	27.72±15.41	26.99±17.71	0.81
**Log sTWEAK (pg/mL)**	2.94±0.28	2.81±0.2	0.005
**Log sCD163 (ng/mL)**	2.3±0.1	2.3±0.1	0.62
**sCD163/sTWEAK**	0.27±0.14	0.34±0.12	0.006
**Log CD14 (ng/mL)**	3.34±0.13	3.18±0.26	<0.001
**Log omentin (ng/mL)**	2.34±0.33	2.09±0.29	<0.001

LDL-cholesterol: low density lipoprotein cholesterol. HDL-cholesterol: High density lipoprotein cholesterol. HOMA-IR: homeostatic model assessment insulin resistance index

Bivariate correlation analysis for the entire cohort with sTWEAK and sCD163 serum levels and clinical, metabolic parameters, biomarkers and immunovirologic status indicated that age, the duration of HIV-1 infection and HAART treatment was not related to sTWEAK or sCD163 levels. No association was observed with any metabolic parameter analyzed (glucose, insulin, HOMA-IR, cholesterol and its fractions, and triglycerides). Concerning immunovirological data, sTWEAK was positively correlated with HIV-1 viral load (r = 0.195; p = 0.042) and sCD163/sTWEAK ratio was negatively correlated with CD4^+^ T-cell count (r = -0.229, p = 0.017) (S1A Table). Bivariate analysis of the subset of HALS patients showed that sTWEAK levels were positively correlated with CD4^+^ T-cell counts (r = 0.312, p = 0.019), while sCD163 levels were positively related with HIV-1 viral load (r = 0.267, p = 0.049). Additionally, sCD163/sTWEAK ratio was negatively correlated with CD4^+^ T-cell counts (r = -0.379, p = 0.004) (S1B Table). Bivariate analysis of the non-HALS group showed that sTWEAK was not associated with any of the parameters analyzed, while sCD163 and sTWEAK/sCD163 ratio were positively correlated with HDL-cholesterol (r = 0.258, p = 0.041, r = 0.275, p = 0.029, respectively) (S1C Table).

The relationships of sTWEAK with inflammatory biomarkers in the entire cohort showed a negative association with sCD163/sTWEAK ratio (r = -0.819, p≤0.001), while this association was positive for log omentin levels and close to significance (r = 0.175, p = 0.056) (S2A Table). On the other hand, log omentin and sCD163/sTWEAK ratio were found positively associated with sCD163 levels (r = 0.317, p≤0.001, r = 0.412, p≤0.001, respectively). When we analyzed HALS patients alone, sTWEAK and sCD163 associated parameters remained the same than for the whole cohort (S2B Table). In non-HALS patients, sTWEAK and SCD163 were found associated with sCD163/sTWEAK ratio (r = -0.830, p≤0.001; r = 0.480, p≤0.001, respectively), and levels of sCD14 and omentin were also found positively associated with sCD163 levels (r = 0.275, p = 0.028; r = 0.400, p = 0.001) (S2C Table).

### Influence of clinical features and antiretroviral treatment on sTWEAK and sCD163 levels

The association between sTWEAK levels and antiretroviral treatment is shown in **Table 3.** We found no differences in sTWEAK levels according to sex, presence of HCV, or NRTI, NNRTI, PI or d4T treatment. Interestingly, AZT treatment was significantly associated with lower sTWEAK levels (p = 0.03). Concerning circulating sCD163, levels were significantly higher in patients with HCV (p≤0.001) and in patients who were on PI treatment (p = 0.002). The ratio sCD163/sTWEAK was found to be significantly associated with the presence of HCV (p = 0.006).

**Table 3:** Influence of antiretroviral treatment on serum clinical characteristics: sTWEAK and sCD163 levels, and ratio sCD163/sTWEAK.

		Log sTweak (pg/mL)	*p* value	Log sCD163 (ng/mL)	*p* value	sCD163/sTWEAK	*p* value
**Sex**	Male	2.87±0.25	0.53	2.3±0.1	0.66	0.31±0.14	0.4
	Female	2.91±0.28		2.29±0.1		0.28±0.12	
**HCV**	Yes	2.86±0.24	0.61	2.36±0.09	<0.001	0.36±0.16	0.006
	No	2.89±0.26		2.28±0.09		0.28±0.12	
**NRTI**	Yes	2.86±0.25	NA	2.3±0.1	NA	0.3±0.13	NA
	No*	3.57		2.35		0.06	
**NNRTI**	Yes	2.86±0.23	0.17	2.29±0.1	0.09	0.31±0.13	0.65
	No	2.95±0.32		2.33±0.09		0.29±0.16	
**PI**	Yes	2.89±0.26	0.12	2.31±0.09	0.002	0.3±0.14	0.77
	No	2.78±0.18		2.22±0.1		0.29±0.09	
**AZT**	Yes	2.83±0.18	0.03	2.29±0.09	0.16	0.32±0.11	0.31
	No	2.96±0.34		2.32±0.1		0.29±0.16	
**d4T**	Yes	2.85±0.21	0.13	2.31±0.09	0.28	0.32±0.13	0.12
	No	2.92±0.31		2.29±0.1		0.28±0.14	

Data are expressed as mean ± standard deviation. HALS: HIV/antiretroviral treatment lipodystrophy syndrome. HCV: hepatitis C virus. NRTI: Nucleoside reverse transcriptase inhibitor. NNRTI: Non-nucleoside reverse transcriptase inhibitor. PI: Protease inhibitor. AZT: zidovudine. d4T: stavudine. NA: Not available.

* Only one patient did not receive NRTI.

### Multivariate analysis

To evaluate the contribution of each variable in determining circulating sTWEAK levels, we constructed a multiple regression model with sTWEAK as dependent variable, and the presence of HALS, AZT treatment, log HIV-1 load, CD4^+^ T-cell count and serum log omentin levels as independent variables. The model had a multiple correlation coefficient of R = 0.397 and indicated that sTWEAK levels were inversely predicted by AZT treatment (B = -0.13, p = 0.02).

A similar analysis was performed for sCD163 levels and included the following variables: HCV, PI treatment, HDL-cholesterol, log HIV-1 load, log omentin and log sCD14. The multivariate analysis showed that levels of sCD163 were predicted positively by the presence of HCV (B = 0.05, p = 0.009), PI treatment (B = 0.07, p = 0.01) and by levels of omentin (B = 0.07, p = 0.04).

Finally, we performed ordered logistic regression analysis to determine the independent predictors of HALS in the whole population. Apart from age and sex, the bivariate associations with HALS observed in univariate analysis were included as independent variables (injection drug user, duration of HAART, log HIV-1 load, d4T treatment, log sTWEAK, log omentin, and log sCD14). Results showed that injection drug users (OR 9.727, 95%CI 1.67–56.7; p = 0.011), lower concentrations of log sTWEAK (OR 0.027, 95%CI 0.001–0.521; p = 0.017), log sCD14 (OR 0.034, 95%CI 0.001–0.775; p = 0.034) and log omentin (OR 0.038, 95%CI 0.004–0.406; p = 0.007) were independently associated with HALS.

## Discussion

The pathogenesis of HALS has been linked with alterations in various metabolic and morphological parameters that are defined by significant changes in the levels of cytokines. A relationship between immune activation and obesity and metabolic disturbances in HALS has previously been established[1].

TWEAK is a cytokine that is emerging as an important mediator of events that occur in chronic inflammatory diseases[33]. Cross-sectional studies have demonstrated that reduced sTWEAK concentrations are associated with type 1 diabetes mellitus (T1DM)[14], (T2DM)[34] and morbid obesity[15]. Additionally, lower sTWEAK concentrations have been related to insulin resistance[15], an increased risk of metabolic syndrome[16] and in general to a poor cardiovascular profile.

We report here for the first time that low sTWEAK concentrations are associated with HALS. A low sTWEAK level emerges as one of the main predictors of a fat redistribution syndrome in HIV-1-infected patients. Interestingly, we found a significant positive association of sTWEAK levels with HIV-1 viral load, indicating a worsening infection profile of HALS patients if sTWEAK levels decrease. Reduced sTWEAK levels have been reported in HIV-1 infected patients, although a 48-week antiretroviral treatment regimen had no effect on the concentrations of sTWEAK[29]. The fact that sTWEAK, in contrast to other cytokines, is reduced rather than elevated in disease conditions associated with chronic inflammatory activity and cardiovascular risk is an unexpected finding and not easily explained. One explanation might be that sTWEAK also has beneficial effects in the regulation of the immune response since TWEAK deficiency in mice leads to the overabundance of natural killer cells and hypersensitivity to bacterial endotoxin, with innate immune cells producing an excess of interferon-γ and IL-12, and less IL-10[6]. TWEAK is pro-apoptotic cytokine[35] and considering that accelerated apoptosis has been detected in subcutaneous adipocytes of HALS patients[36], decreased circulating levels of sTWEAK in these patients might reflect a reduction in sTWEAK due to uptake by its signal transducing receptor Fn14[7], although no data has been published demonstrating this. Other hypotheses could be related to CD163, which can scavenge sTWEAK from the circulation; however in our study, no changes were observed for sCD163 between HALS and non-HALS patients.

HIV-infected patients with high CD4^+^ T-cell counts show a low prevalence of HIV-related conditions[37]. In the present study in HALS patients, higher sTWEAK levels have been found associated with higher CD4^+^ T-cell counts. This may suggest that sTWEAK could have a role as a marker of better immune recovery when on HAART.

Interestingly, we found a positive trend between levels of sTWEAK and levels of omentin, a recently identified insulin-sensitizing adipokine with TNFα inhibitory properties [38,39]. Omentin levels were reduced in HALS patients in this study and in our previous report[2]. This positive association may reinforce the beneficial effects of maintaining sTWEAK levels since low systemic levels of sTWEAK found in other settings, such as severe obesity and T2DM, are associated with an adverse effect on glucose uptake as a consequence of JNK1/2 activation by favoring TNFα signaling[11].

The unique independent predictor of sTWEAK was AZT therapy. AZT treatment has been demonstrated to produce hepatotoxicity due to elevated oxidative and endoplasmic reticulum stress in AZT-treated mice[40]. Additionally, AZT and d4T are among the most pro-inflammatory antiretroviral thymidine analogues[41]; thus, AZT may drive an increase in the production of inflammatory cytokines and, in turn, this could reduce sTWEAK levels. Evidently, in vitro data is necessary to confirm this association.

sCD163 has been associated with the presence or burden of atherosclerotic plaque and arterial wall inflammation in predominantly HIV-monoinfected cohorts[27]. sCD163 levels have been found elevated in HIV patients and are reduced after 48 weeks antiretroviral treatment[29].

In our study, sCD163 levels were not different between HALS and non-HALS patients; it should be noted that both groups were not significantly different regarding antiretroviral treatments. Interestingly, we found higher levels of this macrophage marker in HIV-1-infected patients co-infected with HCV, suggesting a higher inflammatory profile in these patients as shown in other studies[42]. The levels of sCD163 were predicted by the presence of HCV, PI treatment and omentin. A possible compensatory effect of omentin can be attributed to this finding. The concentration of a second monocyte/macrophage marker, sCD14, was increased in non-HALS patients when compared to HALS patients despite viral suppression. These findings are consistent with previous studies supporting the hypothesis that ongoing microbial translocation during treated HIV infection has a dominant role in fueling inflammation in vertically HIV-infected patients[43], and may be contributing to the risk of future cardiovascular disease. sCD163/sTWEAK ratio has been significantly and independently associated with an increased risk for cardiovascular mortality[44]. Here and in previous studies, sCD163/sTWEAK ratio was found inversely correlated with CD4 counts[29]. Along this line, our study also found that sCD163/sTWEAK ratio was the only independent predictor of HALS, reinforcing the value of both biomarkers in HALS pathology.

We are aware that the cross-sectional design of this study precludes conclusions about causality and about the biological significance of sTWEAK in this context. The answer as to which pathophysiologic mechanisms could explain the association between lower sTWEAK levels and HALS will need further investigation. Of note, measurements of IL-10 (and also of IL-6) could possibly have provided some insight regarding the mechanism by which sCD163 exerts its anti-inflammatory properties but, as commented above, unfortunately no additional plasma samples were available to perform these measurements.

In summary, we found that HALS is associated with decreased sTWEAK levels. Furthermore, both sTWEAK levels and sCD163/sTWEAK ratio could be considered biomarkers in patients with HALS pathology, although we acknowledge that further cohort validation is required for a generalization of these results.

## Supporting Information

S1 Table Correlation between serum sTWEAK, sCD163 and sCD163/sTWEAK ratio and immunovirological and metabolic parameters. (DOCX)

S2 Table Relationship between serum sTWEAK, serum sCD163, CD163/TWEAK ratio and other cytokines. (DOCX)
