# Supplementary material for: HIV-1/HAART-Related Lipodystrophy Syndrome (HALS) Is Associated with Decreased Circulating sTWEAK Levels
Source: PLoS One. 2015 Dec 14;10(12):e0144789. doi: 10.1371/journal.pone.0144789 (PMC4684375; doi:10.1371/journal.pone.0144789)
Supplement: S1 Table — (DOCX) [file pone.0144789.s001.docx]

**Correlation between serum sTWEAK, sCD163 and sCD163/sTWEAK ratio and immunovirological and metabolic parameters**

**Table A**

| **The whole series** |  | **Log sTWEAK (pg/mL)** | **Log sCD163 (ng/mL)** | **sCD163/sTWEAK ratio** |
| --- | --- | --- | --- | --- |
| **Age (years)** | r | -0.071 | 0.097 | 0.153 |
|  | p | 0.439 | 0.294 | 0.094 |
| **HIV duration (years)** | r | -0.102 | 0.090 | 0.155 |
|  | p | 0.278 | 0.337 | 0.098 |
| **HAART duration (months)** | r | -0.156 | -0.105 | 0.139 |
|  | p | 0.090 | 0.255 | 0.130 |
| **Glucose (mmol/L)** | r | 0.006 | 0.036 | 0.023 |
|  | p | 0.951 | 0.694 | 0.800 |
| **Insulin (µU/mL)** | r | -0.049 | -0.049 | 0.043 |
|  | p | 0.598 | 0.597 | 0.643 |
| **HOMA-IR** | r | -0.015 | -0.003 | 0.018 |
|  | p | 0.870 | 0.976 | 0.842 |
| **Cholesterol (mmol/L)** | r | -0.046 | -0.078 | 0.013 |
|  | p | 0.617 | 0.402 | 0.891 |
| **HDL cholesterol (mmol/L)** | r | -0.067 | 0.139 | 0.122 |
|  | p | 0.470 | 0.131 | 0.186 |
| **LDL cholesterol (mmol/L)** | r | -0.044 | -0.087 | -0.004 |
|  | p | 0.633 | 0.345 | 0.962 |
| **Triglycerides (mmol/L)** | r | 0.067 | -0.166 | -0.101 |
|  | p | 0.469 | 0.072 | 0.275 |
| **Log HIV viral load (copies/mL)** | r | 0.195 | 0.060 | -0.161 |
|  | p | 0.042 | 0.532 | 0.092 |
| **CD4 cell count (cells/mL)** | r | 0.110 | -0.064 | -0.229 |
|  | p | 0.254 | 0.506 | 0.017 |

**Table B**

| **HALS** |  | **Log sTWEAK (pg/mL)** | **Log sCD163 (ng/ml)** | **sCD163/sTWEAK ratio** |
| --- | --- | --- | --- | --- |
| **Age (years)** | r | 0.014 | 0.089 | 0.131 |
|  | p | 0.919 | 0.514 | 0.335 |
| **HIV duration (years)** | r | 0.006 | .271^*^ | 0.146 |
|  | p | 0.965 | 0.048 | 0.293 |
| **HAART duration (months)** | r | -0.225 | -0.102 | 0.187 |
|  | p | 0.096 | 0.453 | 0.167 |
| **Glucose (mmol/L)** | r | -0.058 | -0.035 | 0.018 |
|  | p | 0.674 | 0.799 | 0.897 |
| **Insulin (µU/mL)** | r | -0.049 | -0.097 | 0.047 |
|  | p | 0.720 | 0.477 | 0.731 |
| **HOMA-IR** | r | -0.022 | -0.043 | -0.002 |
|  | p | 0.870 | 0.755 | 0.989 |
| **Cholesterol (mmol/L)** | r | 0.072 | -0.098 | -0.130 |
|  | p | 0.600 | 0.473 | 0.341 |
| **HDL cholesterol (mmol/L)** | r | 0.034 | 0.010 | -0.052 |
|  | p | 0.802 | 0.941 | 0.703 |
| **LDL cholesterol (mmol/L)** | r | -0.006 | -0.119 | -0.093 |
|  | p | 0.967 | 0.381 | 0.495 |
| **Triglycerides (mmol/L)** | r | 0.006 | -0.206 | -0.138 |
|  | p | 0.963 | 0.128 | 0.309 |
| **Log HIV viral load (copies/mL)** | r | 0.206 | 0.267 | -0.099 |
|  | p | 0.132 | 0.049 | 0.473 |
| **CD4 cell count (cells/mL)** | r | 0.312 | -0.073 | -0.379 |
|  | p | 0.019 | 0.594 | 0.004 |

**Table C**

| **Non-HALS** |  | **Log sTWEAK (pg/mL)** | **Log sCD163 (ng/mL)** | **sCD163/sTWEAK ratio** |
| --- | --- | --- | --- | --- |
| **Age (years)** | r | -0.059 | 0.092 | 0.110 |
|  | p | 0.641 | 0.472 | 0.386 |
| **HIV duration (years)** | r | -0.066 | -0.093 | 0.056 |
|  | p | 0.611 | 0.478 | 0.669 |
| **HAART duration (months)** | r | -0.097 | -0.114 | 0.077 |
|  | p | 0.446 | 0.368 | 0.546 |
| **Glucose (mmol/L)** | r | 0.038 | 0.098 | 0.041 |
|  | p | 0.767 | 0.444 | 0.751 |
| **Insulin (µU/mL)** | r | -0.064 | -0.002 | 0.053 |
|  | p | 0.618 | 0.985 | 0.676 |
| **HOMA-IR** | r | 0.025 | 0.064 | 0.025 |
|  | p | 0.848 | 0.617 | 0.844 |
| **Cholesterol (mmol/L)** | r | -0.091 | -0.072 | 0.091 |
|  | p | 0.480 | 0.576 | 0.476 |
| **HDL-cholesterol (mmol/L)** | r | -0.132 | 0.258 | 0.275 |
|  | p | 0.301 | 0.041 | 0.029 |
| **LDL-cholesterol (mmol/L)** | r | -0.037 | -0.069 | 0.033 |
|  | p | 0.775 | 0.589 | 0.797 |
| **Triglycerides (mmol/L)** | r | 0.170 | -0.139 | -0.114 |
|  | p | 0.184 | 0.278 | 0.372 |
| **Log HIV viral load (copies/mL)** | r | 0.102 | 0.015 | -0.085 |
|  | p | 0.457 | 0.913 | 0.537 |
| **CD4 cell count (cells/mL)** | r | -0.156 | -0.019 | 0.049 |
|  | p | 0.265 | 0.893 | 0.730 |
