# Supplementary material for: HIV-1/HAART-Related Lipodystrophy Syndrome (HALS) Is Associated with Decreased Circulating sTWEAK Levels
Source: PLoS One. 2015 Dec 14;10(12):e0144789. doi: 10.1371/journal.pone.0144789 (PMC4684375; doi:10.1371/journal.pone.0144789)
Supplement: S2 Table — (DOCX) [file pone.0144789.s002.docx]

**Relationship between serum sTWEAK, serum sCD163, CD163/TWEAK ratio and other cytokines**

**Table A**

| **The whole series** |  | **Log sTWEAK (pg/mL)** | **Log sCD163**  **(ng/mL)** | **sCD163/sTWEAK ratio** |
| --- | --- | --- | --- | --- |
| **Log sCD163 (ng/mL)** | r | 0.056 |  |  |
|  | p | 0.540 |  |  |
| **sCD163/sTWEAK ratio** | r | -0.819 | 0.412 |  |
|  | p | <0.001 | <0.001 |  |
| **Log omentin (ng/mL)** | r | 0.175 | 0.317 | 0.037 |
|  | p | 0.056 | <0.001 | 0.692 |
| **Log sCD14 (ng/mL)** | r | 0.022 | 0.067 | 0.057 |
|  | p | 0.809 | 0.468 | 0.535 |

**Table B**

| **HALS** |  | **Log sTWEAK (pg/mL)** | **Log sCD163 (ng/mL)** | **sCD163/sTWEAK ratio** |
| --- | --- | --- | --- | --- |
| **Log sCD163 (ng/mL)** | r | 0.257 |  |  |
|  | p | 0.056 |  |  |
| **sCD163/sTWEAK ratio** | r | -0.782 | 0.325 |  |
|  | p | <0.001 | 0.015 |  |
| **Log omentin (ng/mL)** | r | 0.088 | 0.302 | 0.086 |
|  | p | 0.518 | 0.024 | 0.527 |

**Table C**

| **Non-HALS** |  | **Log sTWEAK (pg/mL)** | **Log sCD163**  **(ng/mL)** | **sCD163/sTWEAK**  **ratio** |
| --- | --- | --- | --- | --- |
| **Log sCD163 (ng/mL)** | r | -0.037 |  |  |
|  | p | 0.770 |  |  |
| **sCD163/sTWEAK ratio** | r | -0.830 | 0.480 |  |
|  | p | <0.001 | <0.001 |  |
| **Log omentin (ng/mL)** | r | 0.095 | 0.400 | 0.179 |
|  | p | 0.453 | 0.001 | 0.156 |
| **Log sCD14 (ng/mL)** | r | -0.059 | 0.275 | 0.222 |
|  | p | 0.643 | 0.028 | 0.078 |
